# Glycemic biomarkers and cardiovascular disease in diabetes: a comparative analysis of population-based and clinical cohorts

**DOI:** 10.3389/fendo.2026.1995413

**Published:** 2026-10-14

**Authors:** Lihong Zhang, Meng Liu, Yu Zhang, Liyuan Zhao, Yunjiang Liu, Rui Wang, Juan Zhou, Kun Li

**Affiliations:** 1 Clinical Laboratory, Affiliated Hospital of West Anhui Health Vocational College, Lu’an, China; 2 Department of Clinical Pharmacy, Affiliated Hospital of West Anhui Health Vocational College, Lu’an, China

**Keywords:** cardiovascular disease, diabetes, glycated hemoglobin A1c, glycemic control, triglyceride-glucose index

## Abstract

**Background:**

Cardiovascular disease (CVD) remains a major complication contributing to morbidity and mortality among individuals with diabetes. Different glycemic biomarkers reflect distinct aspects of glucose metabolism, but their associations with prevalent CVD across different diabetic populations remain incompletely understood. This study aimed to evaluate the associations of fasting blood glucose (FBG), glycated hemoglobin A1c (HbA1c), and triglyceride-glucose index (TyG) with prevalent CVD among adults with diabetes.

**Methods:**

This study included adults with diabetes from two independent cross-sectional cohorts: a population-based cohort derived from the National Health and Nutrition Examination Survey (NHANES) 2005–2020 (n = 3,418) and a Chinese clinical cohort of hospitalized patients with diabetes (n = 765). Multivariable logistic regression models, subgroup analyses, and restricted cubic spline (RCS) analyses were performed to evaluate the associations between glycemic biomarkers and prevalent CVD.

**Results:**

After adjustment for potential confounders, HbA1c was consistently associated with prevalent CVD in both cohorts. In the fully adjusted model, each unit increase in HbA1c was associated with higher odds of prevalent CVD in the population-based cohort (OR = 1.097, 95% CI: 1.002–1.202, *P* = 0.045) and the clinical cohort (OR = 1.413, 95% CI: 1.280–1.564, *P* < 0.001). In contrast, FBG and TyG were not significantly associated with prevalent CVD in the fully adjusted models. RCS analyses showed a significant overall association between HbA1c and prevalent CVD without evidence of nonlinearity in the NHANES cohort, whereas a significant nonlinear association was observed in the clinical cohort. TyG also showed a significant nonlinear association with prevalent CVD in the NHANES cohort. Subgroup analyses did not show consistent evidence of effect modification across demographic and clinical characteristics.

**Conclusion:**

Among adults with diabetes, HbA1c showed a consistent positive association with prevalent CVD across population-based and clinical cohorts, whereas the associations of FBG and TyG were less consistent. These findings support the relevance of HbA1c as a measure of long-term glycemic status in relation to prevalent CVD among individuals with diabetes. Further longitudinal studies are warranted to clarify the temporal relationship between HbA1c and cardiovascular outcomes.

## 1 Introduction

With global population growth and accelerated aging, the prevalence of diabetes has increased sharply (1). Cardiovascular disease (CVD) is the leading cause of mortality and disability among individuals with diabetes (2, 3). Epidemiological evidence shows that adults with diabetes have a 2- to 4-fold higher risk of developing CVD compared to non-diabetic individuals, and this risk is closely linked to glycemic control (4, 5). Although advances in glucose-lowering therapies have improved cardiovascular outcomes in selected populations, accurately characterizing CVD burden among individuals with diabetes remains a major clinical challenge (6). Therefore, identifying clinically relevant biomarkers that reflect different aspects of glycemic status may provide valuable insights into the relationship between glycemic control and CVD and inform diabetes management.

Poor glycemic control is considered an important contributor to cardiovascular complications in individuals with diabetes mellitus. Studies have shown significant associations between elevated glycated hemoglobin A1c (HbA1c) levels and increased CVD risk (7). Although the complexity of metabolic abnormalities in diabetes complicates the understanding of its role in macrovascular disease, hyperglycemia-related processes are well recognized as important mechanisms involved in macrovascular complications (8). Chronic hyperglycemia and insulin resistance contribute to endothelial dysfunction, oxidative stress, and vascular injury, thereby accelerating cardiovascular complications in diabetes (9, 10). Clinical studies further support the link between glycemic control and CVD events: poorer glycemic control is associated with a higher burden of cardiovascular complications (11, 12). A meta-analysis of 102 prospective studies involving 698,782 participants found that fasting plasma glucose levels ≥7 mmol/L were associated with a higher risk of coronary heart disease (13). Furthermore, longer diabetes duration and inadequate glycemic control are significantly associated with increased risks of CVD and all-cause mortality (14, 15).

Several glycemic biomarkers are currently available to characterize glucose metabolism, but they reflect distinct biological dimensions. Fasting blood glucose (FBG), HbA1c, and the triglyceride-glucose index (TyG) represent different aspects of glycemic status, including short-term glucose levels, long-term glycemic exposure, and insulin resistance, respectively. Previous studies have demonstrated a strong association between elevated FBG levels and increased CVD risk (16), with increases in FBG within the normal range also associated with higher CVD risk (17). A Swiss cohort study involving 6,095 participants found that FBG could serve as a substitute for total cholesterol in predicting CVD mortality risk (18). Additionally, analyses from a Japanese epidemiological database highlighted the importance of maintaining optimal FBG levels for the primary prevention of CVD in young adults (19). HbA1c, a marker of long-term glycemic control, is significantly associated with increased risks of CVD and mortality in diabetic patients when poorly controlled (20). Studies have reported a positive correlation between baseline HbA1c levels and the severity of coronary artery disease (21). Moreover, insulin resistance is a major risk factor for CVD (22), and TyG, a widely used surrogate marker of insulin resistance, has been extensively investigated in relation to cardiovascular outcomes (23, 24).

Although individual glycemic biomarkers have been associated with cardiovascular outcomes, comparative evaluations of multiple indicators reflecting short-term glucose levels, long-term glycemic exposure, and insulin resistance remain limited. Whether these biomarkers show consistent associations with prevalent CVD among individuals with diabetes remains unclear.

In this study, we systematically investigated the associations between glycemic biomarkers and prevalent CVD using data from the 2005–2020 National Health and Nutrition Examination Survey (NHANES) and an independent clinical diabetes cohort. We conducted a comparative analysis of FBG, HbA1c, and TyG in two independent cross-sectional cohorts, comprising a population-based cohort and a clinically characterized cohort. Our aim was to characterize the associations of these complementary glycemic biomarkers with prevalent CVD and to assess the consistency of these associations across the two study populations.

## 2 Methods

### 2.1 Study design and participants

This study was designed as a comparative analysis of two independent cross-sectional cohorts, including a population-based cohort from the NHANES and a clinically characterized diabetes cohort from China. The aim was to evaluate the associations between different glycemic biomarkers and prevalent CVD among individuals with diabetes across different study settings.

NHANES is a continuous population-based survey conducted in two-year cycles in the United States to assess the health and nutritional status of the noninstitutionalized U.S. population using a complex, multistage probability sampling design. In this study, participants from seven NHANES cycles (2005–2006, 2007–2008, 2009–2010, 2011–2012, 2013–2014, 2015–2016, and 2017–March 2020) were considered for inclusion. The 2017–March 2020 cycle represents the prepandemic release. Participants with available information on diabetes status, glycemic biomarkers, prevalent CVD, and relevant covariates were considered for the final analysis. Diabetes was defined as meeting at least one of the following criteria: a physician-diagnosed diabetes; HbA1c ≥6.5%; FBG ≥7.0 mmol/L; random plasma glucose ≥11.1 mmol/L; 2-hour postprandial glucose (2hPG) ≥11.1 mmol/L; or current use of antidiabetic medication or insulin (25). After excluding individuals with missing data, a total of 3,418 participants were included in the final analysis. Detailed information is presented in **Figure 1**.

**Figure 1:**
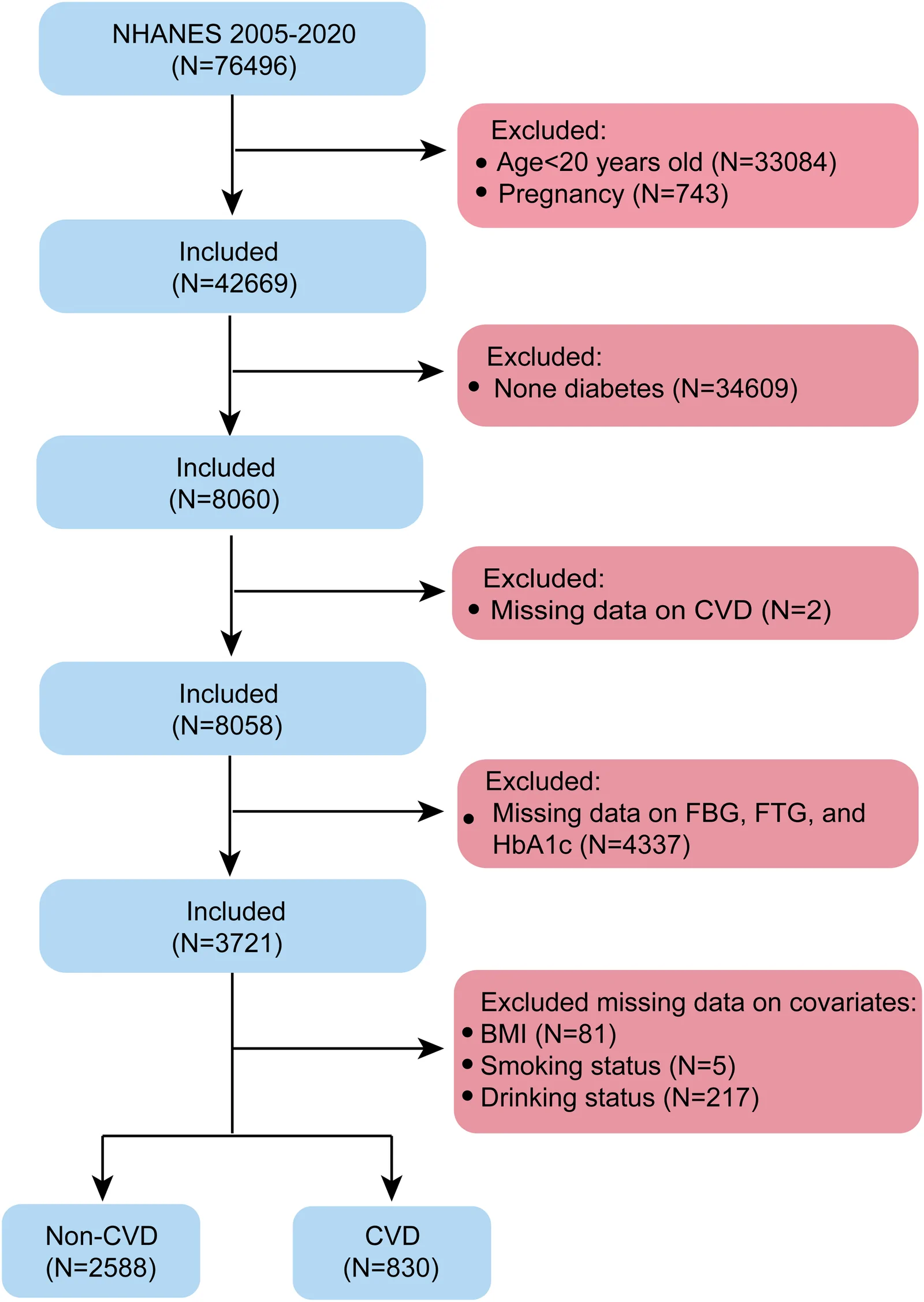
Flowchart of participant inclusion.

To account for the complex multistage sampling design of NHANES, all analyses incorporated the appropriate survey weights, strata, and primary sampling units. Because the analyses included fasting laboratory measurements, fasting subsample examination weights were used. For pooled analyses across survey cycles, the fasting subsample weights were rescaled according to the NHANES analytic guidelines, with the contribution of each survey period adjusted according to its respective survey duration. This approach was used to obtain estimates representative of the target U.S. population. The analytical sample was restricted to participants who met the fasting requirements and had available biomarker and covariate data. This restriction was necessary because FBG and triglyceride concentrations can be influenced by recent food intake.

The clinical diabetes cohort consisted of 765 adult patients with diabetes who were consecutively recruited from the Department of Endocrinology at the Affiliated Hospital of West Anhui Health Vocational College between January 2024 and April 2025. Diabetes was defined using established clinical diagnostic criteria consistent with those recommended by the American Diabetes Association (25).

The inclusion criteria for the clinical cohort were (1): age ≥18 years (2); confirmed diagnosis of diabetes mellitus; and (3) availability of complete clinical information and laboratory measurements, including glucose-related parameters and cardiovascular-related assessments. The exclusion criteria were (1): pregnancy (2); malignant tumors or history of cancer treatment (3); acute infectious diseases, severe inflammatory conditions, or acute metabolic complications at enrollment (4); severe hepatic or renal dysfunction (5); autoimmune diseases or hematological disorders that may affect metabolic parameters; and (6) incomplete clinical or laboratory data.

All participants in the clinical cohort underwent standardized clinical evaluation, and fasting venous blood samples were collected for biochemical measurements. Clinical information and laboratory measurements were obtained at the time of enrollment, and CVD status was determined from clinical records available at that time.

### 2.2 Exposure variables

This study employed three glycemic biomarkers, including FBG, HbA1c, and TyG, as exposure variables. In NHANES, fasting blood samples were collected at the Mobile Examination Center (MEC) and processed according to standardized NHANES laboratory procedures. In the clinical cohort, fasting venous blood samples were collected after at least 8 hours of fasting and immediately analyzed in the hospital clinical laboratory using standardized procedures. FBG and triglycerides (TG) levels were measured using enzymatic methods in both cohorts, and HbA1c was determined using standardized high-performance liquid chromatography (HPLC)-based assays. The TyG index was calculated as ln[FBG (mg/dL) × TG (mg/dL)/2].

### 2.3 Outcome variables

The primary outcome of this study was prevalent CVD. To facilitate comparability between the two cohorts, CVD was defined based on the presence of at least one major cardiovascular condition, including congestive heart failure, coronary heart disease, angina, myocardial infarction, or stroke. However, the ascertainment methods differed between the two cohorts. In NHANES, CVD status was ascertained using standardized medical questionnaires. Participants were classified as having CVD if they self-reported a previous physician diagnosis of congestive heart failure, coronary heart disease, angina, myocardial infarction, or stroke (26).

In the clinical diabetes cohort, CVD status was determined from documented medical records and clinical diagnoses available at the time of enrollment. Patients were considered to have CVD if they had documented congestive heart failure, coronary heart disease, angina pectoris, myocardial infarction, or stroke.

### 2.4 Covariates

Potential confounding factors associated with glycemic status and CVD were considered in the multivariable analyses. Covariates were selected *a priori* based on clinical relevance, previous epidemiological evidence, and their potential to confound the associations between glycemic biomarkers and prevalent CVD. These included demographic characteristics, lifestyle factors, adiposity, hypertension, lipid profiles, renal function, hemoglobin concentration, and diabetes- and cardiovascular-related medication use. Specifically, age, sex, smoking status, drinking status, body mass index (BMI), hypertension status, total cholesterol (TC), high-density lipoprotein cholesterol (HDL-C), low-density lipoprotein cholesterol (LDL-C), estimated glomerular filtration rate (eGFR), hemoglobin (Hb), glucose-lowering medication use, insulin use, lipid-lowering medication use, and antihypertensive medication use were included as covariates. Diabetes duration was additionally included in the clinical cohort.

Age was categorized into four groups (20–39, 40–59, 60–79, and ≥80 years), and sex was categorized as female or male. Lifestyle factors included smoking status (non-smoker: <100 cigarettes in lifetime; smoker: ≥100 cigarettes) and drinking status (non-drinker: <12 alcoholic drinks per year; drinker: ≥12 drinks) (27, 28). BMI was categorized as <25, 25 to <30, and ≥30 kg/m² for subgroup analyses and was analyzed as a continuous variable in the multivariable regression models. Hypertension was defined as an average blood pressure ≥140/90 mmHg based on repeated blood pressure measurements or a previous physician diagnosis of hypertension.

Covariate availability differed between the two cohorts. In the NHANES cohort, race/ethnicity and the poverty-to-income ratio (PIR) were additionally considered as demographic and socioeconomic covariates. Diabetes duration was not included in the NHANES models because a reliable date of diabetes diagnosis was not available for all participants, particularly those identified based on laboratory criteria. In the clinical cohort, diabetes duration was obtained from patient medical histories and medical records and was therefore included in the corresponding models. Race/ethnicity was not included in the clinical cohort because all participants were Han Chinese, and PIR was not available because standardized socioeconomic information was not routinely collected. Medication use in NHANES was ascertained from standardized questionnaire data, whereas medication use in the clinical cohort was obtained from documented medical records.

### 2.5 Statistical analysis

Baseline characteristics were summarized as means (standard error) for continuous variables and counts (percentages) for categorical variables. Continuous variables were compared between participants with and without CVD using independent-samples *t* tests, and categorical variables were compared using the chi-square test. Multivariable logistic regression models were constructed to evaluate the associations between glycemic biomarkers and prevalent CVD among adults with diabetes. Model 1 was an unadjusted model. Model 2 was adjusted for age and sex, with additional adjustment for race/ethnicity and the PIR in the NHANES cohort. Model 3 was further adjusted for smoking status, drinking status, BMI, and hypertension. Model 4 was additionally adjusted for TC, HDL-C, LDL-C, eGFR, glucose-lowering medication use, insulin use, lipid-lowering medication use, antihypertensive medication use, and Hb. Diabetes duration was additionally included in Model 4 for the clinical cohort. Race/ethnicity and PIR were not included in the clinical cohort models.

To facilitate comparison of the associations across the three glycemic biomarkers, standardized ORs were estimated per 1-standard-deviation (SD) increase in FBG, HbA1c, and TyG using the fully adjusted Model 4. Standardization was performed separately within each cohort based on the overall study population rather than by CVD status. Subgroup analyses were performed for HbA1c in both cohorts, and potential effect modification was assessed using multiplicative interaction terms. *P* values for interaction were reported. Restricted cubic spline (RCS) analyses were performed to examine potential nonlinear associations between FBG, HbA1c, TyG, and prevalent CVD after adjustment for all covariates in Model 4. Three knots were specified for each biomarker, with knot locations determined within the RCS model. The median concentration of each biomarker was used as the reference value. Overall associations and departures from linearity were evaluated using the corresponding model-based *P* values.

All statistical analyses were conducted using R software (version 4.4.0). Data visualization was performed using the ggplot2 and forestploter packages. A two-sided *P* value <0.05 was considered statistically significant.

## 3 Results

### 3.1 Baseline characteristics

This study included two independent cross-sectional cohorts of adults with diabetes. The NHANES cohort consisted of 3,418 participants, including 2,588 individuals without CVD and 830 individuals with CVD, corresponding to a prevalence of 24.3%. The clinical cohort included 765 hospitalized patients with diabetes, among whom 467 were classified as non-CVD and 298 as having CVD, corresponding to a prevalence of 39.0%.

In both cohorts, participants with CVD were generally older and had a higher prevalence of hypertension than those without CVD. Lipid profiles, renal function, hemoglobin levels, and medication use were also assessed. Detailed baseline characteristics of the two cohorts are presented in **Table 1**.

**Table 1:** Baseline characteristics of the study populations.

Characteristic	NHANES		Clinical cohort	
	Non-CVD (n=2588)	CVD (n=830)	Non-CVD (n=467)	CVD (n=298)
Age, years, n (%)				
20-39	246 (11.65)	12 (2.19)	47 (10.06)	1 (0.34)
40-59	926 (41.71)	156 (20.74)	252 (53.96)	102 (34.23)
60-79	1206 (40.02)	508 (59.91)	163 (34.90)	174 (58.39)
≥80	210 (6.62)	154 (17.15)	5 (1.07)	21 (7.05)
Sex, n (%)				
Female	1297 (52.03)	356 (41.94)	218 (46.68)	150 (50.34)
Male	1291 (47.97)	474 (58.06)	249 (53.32)	148 (49.66)
Race/ethnicity, n (%) , n (%)				
Non-Hispanic White	897(61.07)	405(71.34)	–	–
Non-Hispanic Black	614(13.46)	191(12.95)	–	–
Mexican American	502(10.92)	98(5.31)	–	–
Other race	575(14.56)	136(10.41)	–	–
BMI (kg/m^2^), n (%)				
<25	373 (12.08)	97 (11.98)	196 (41.97)	136 (45.64)
25 to <30	767 (27.63)	242 (27.86)	221 (47.32)	134 (44.97)
≥30	1448 (60.29)	491 (60.16)	50 (10.71)	28 (9.40)
Smoking status, n (%)				
No	1387 (53.73)	353 (40.31)	358 (76.66)	224 (75.17)
Yes	1201 (46.27)	477 (59.69)	109 (23.34)	74 (24.83)
Drinking status, n (%)				
No	427 (14.64)	133 (14.02)	363 (77.73)	235 (78.86)
Yes	2161 (85.36)	697 (85.98)	104 (22.27)	63 (21.14)
Hypertension, n (%)				
No	829 (32.35)	132 (17.46)	244 (52.25)	106 (35.57)
Yes	1759 (67.65)	698 (82.54)	223 (47.75)	192 (64.43)
Glucose-lowering medication use, n (%)				
No	1328 (51.44)	390 (46.09)	40 (8.57)	11 (3.69)
Yes	1260 (48.56)	440 (53.91)	427 (91.43)	287 (96.31)
Insulin use, n (%)				
No	2430 (94.00)	725 (89.07)	221 (47.32)	138 (46.31)
Yes	158 (6.00)	105 (10.93)	246 (52.68)	160 (53.69)
Lipid-lowering medication use, n (%)				
No	1487 (56.96)	242 (24.38)	311 (66.60)	118 (39.60)
Yes	1101 (43.04)	588 (75.62)	156 (33.40)	180 (60.40)
Antihypertensive medication use, n (%)				
No	1017 (39.81)	97 (11.47)	278 (59.53)	125 (41.95)
Yes	1571 (60.19)	733 (88.53)	189 (40.47)	173 (58.05)
PIR, mean (SE)	2.81 (0.05)	2.56 (0.09)	–	–
Diabetes duration, years, mean (SE)	–	–	7.48 (0.28)	10.48 (0.41)
FBG (mmol/L), mean (SE)	8.24 (0.08)	8.19 (0.15)	11.19 (0.19)	11.81 (0.26)
TC (mmol/L), mean (SE)	4.88(0.04)	4.47(0.06)	4.98 (0.06)	4.60 (0.07)
TG (mmol/L), mean (SE)	1.84 (0.05)	1.81 (0.07)	2.27 (0.12)	2.07 (0.10)
HDL-C (mmol/L), mean (SE)	1.28(0.01)	1.21(0.02)	1.34 (0.02)	1.31 (0.02)
LDL-C (mmol/L), mean (SE)	2.80(0.03)	2.45(0.05)	3.08 (0.05)	2.83 (0.06)
Hb (g/L), mean (SE)	142.02(0.43)	141.07(0.86)	138.77 (0.87)	132.40 (1.14)
HbA1c (%), mean (SE)	6.95 (0.04)	7.05 (0.09)	9.11 (0.09)	10.36 (0.10)
TyG, mean (SE)	9.14 (0.02)	9.14 (0.04)	9.58 (0.03)	9.58 (0.04)
eGFR (mL/min/1.73 m²), mean (SE)	90.10(0.60)	75.43(1.13)	104.04 (1.00)	91.06 (1.33)

NHANES sample counts are unweighted, whereas percentages for categorical variables and summary estimates for continuous variables are survey-weighted to account for the complex survey design. CVD, cardiovascular disease; BMI, body mass index; PIR, poverty-to-income ratio; FBG, fasting blood glucose; TC, total cholesterol; TG, triglycerides; HDL-C, high-density lipoprotein cholesterol; LDL-C, low-density lipoprotein cholesterol; Hb, hemoglobin; HbA1c, glycated hemoglobin A1c; TyG, triglyceride-glucose index; eGFR, estimated glomerular filtration rate.

### 3.2 Association between glycemic biomarkers and CVD in patients with diabetes

In the NHANES cohort, HbA1c showed a positive association with prevalent CVD after multivariable adjustment. The association remained statistically significant in Model 2 and Model 3 and was attenuated but remained significant after further adjustment for metabolic, renal, hematological, and medication-related covariates in Model 4. In contrast, FBG and TyG were not significantly associated with CVD in the fully adjusted model. Similar findings were observed in the clinical cohort, in which HbA1c was consistently positively associated with CVD across the multivariable models, whereas FBG and TyG were not significantly associated with CVD in Model 4. In Model 4, each unit increase in HbA1c was associated with higher odds of prevalent CVD in both the NHANES cohort (OR, 1.097; 95% CI, 1.002–1.202; *P* = 0.045) and the clinical cohort (OR, 1.413; 95% CI, 1.280–1.564; *P* < 0.001) (**Table 2**).

**Table 2:** Association between glycemic biomarkers and prevalent CVD among adults with diabetes.

Model	NHANES		Clinical cohort	
	OR (95%CI)	*P*	OR (95%CI)	*P*
Model 1				
FBG	0.995 (0.962, 1.029)	0.776	1.033 (0.999, 1.069)	0.059
HbA1c	1.037 (0.974, 1.104)	0.254	1.437 (1.322, 1.567)	<0.001
TyG	0.999 (0.868, 1.151)	0.992	1.008 (0.824, 1.231)	0.941
Model 2				
FBG	1.023 (0.984, 1.064)	0.254	1.036 (0.998, 1.076)	0.062
HbA1c	1.115 (1.034, 1.202)	0.005	1.426 (1.302, 1.566)	<0.001
TyG	1.157 (0.976, 1.371)	0.092	0.997 (0.798, 1.241)	0.976
Model 3				
FBG	1.023 (0.983, 1.065)	0.258	1.035 (0.997, 1.074)	0.071
HbA1c	1.117 (1.038, 1.203)	0.004	1.429 (1.307, 1.568)	<0.001
TyG	1.099 (0.924, 1.306)	0.283	0.989 (0.782, 1.231)	0.919
Model 4				
FBG	1.023 (0.979, 1.069)	0.310	1.031 (0.991, 1.072)	0.136
HbA1c	1.097 (1.002, 1.202)	0.045	1.413 (1.280, 1.564)	<0.001
TyG	0.975 (0.668, 1.423)	0.894	0.959 (0.757, 1.210)	0.723

Model 1 included no covariate adjustments. Model 2 was adjusted for age and sex, with additional adjustment for Race and PIR in the NHANES cohort. Model 3 was further adjusted for smoking status, drinking status, BMI, and hypertension. Model 4 was further adjusted for TC, HDL-C, LDL-C, eGFR, glucose-lowering medication use, insulin use, lipid-lowering medication use, antihypertensive medication use, and Hb. In the clinical cohort, Model 4 additionally included diabetes duration. Race/ethnicity and PIR were not included in the clinical cohort models because the clinical cohort consisted exclusively of Han Chinese participants and did not have standardized socioeconomic data. CVD, cardiovascular disease; FBG, fasting blood glucose; HbA1c, glycated hemoglobin A1c; TyG, triglyceride-glucose index; PIR, poverty-to-income ratio; BMI, body mass index; TC, total cholesterol; HDL-C, high-density lipoprotein cholesterol; LDL-C, low-density lipoprotein cholesterol; eGFR, estimated glomerular filtration rate; Hb, hemoglobin.

To facilitate direct comparison across the three glycemic biomarkers, standardized associations were additionally estimated per 1-SD increase using the fully adjusted model. The results were consistent with the primary analyses, with HbA1c remaining significantly associated with prevalent CVD in both the NHANES cohort (OR, 1.175; 95% CI, 1.004–1.376; *P* = 0.045) and the clinical cohort (OR, 1.944; 95% CI, 1.608–2.364; *P* < 0.001), whereas FBG and TyG showed no statistically significant associations in either cohort (**Supplementary Table 1**).

### 3.3 Subgroup analyses

Subgroup analyses were performed to further evaluate whether the association between HbA1c and prevalent CVD varied across different demographic and clinical characteristics in the two cohorts (**Figures 2**, **3**). In the NHANES cohort, the positive association between HbA1c and prevalent CVD was statistically significant among participants aged 60–79 years (OR, 1.139; 95% CI, 1.025–1.265; *P* = 0.016); however, no significant interaction was observed between HbA1c and age group (*P* for interaction=0.927). A significant interaction was observed for drinking status (*P* for interaction=0.031), although the associations were not statistically significant within either drinking subgroup. No significant interactions were observed for sex, BMI category, smoking status, or hypertension (all *P* for interaction>0.05).

**Figure 2:**
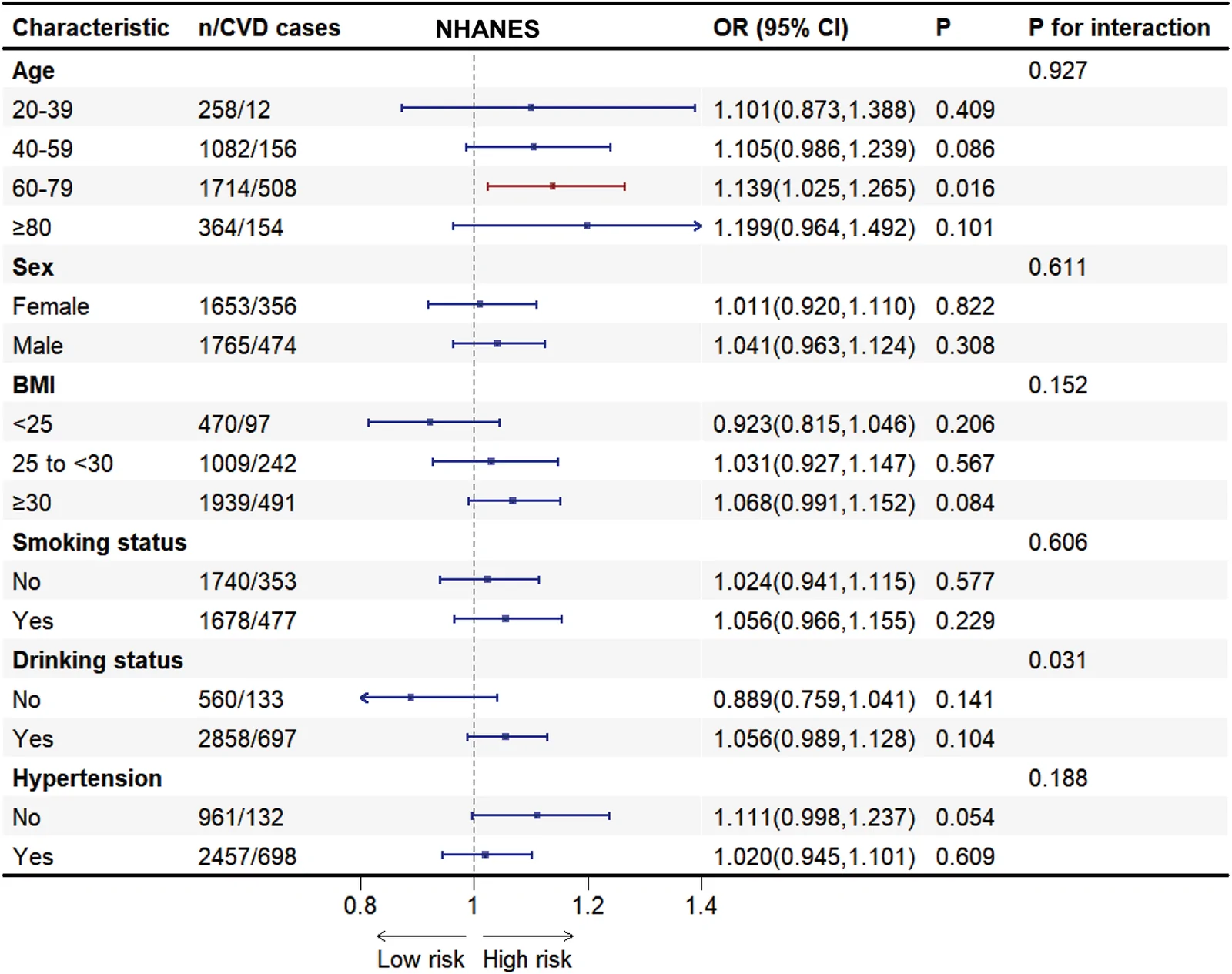
Subgroup analysis of the association between HbA1c and prevalent CVD among adults with diabetes in the NHANES cohort.

**Figure 3:**
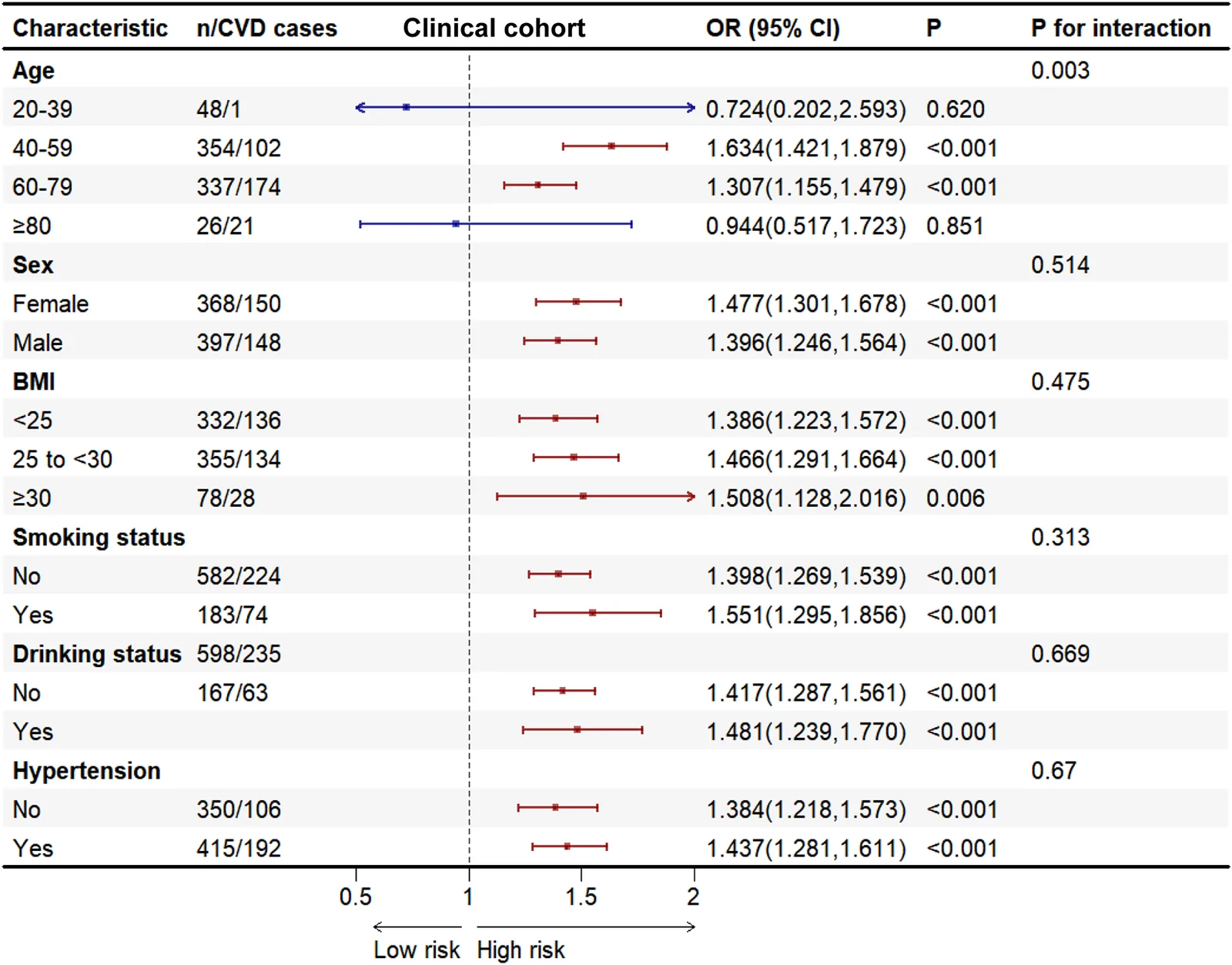
Subgroup analysis of the association between HbA1c and prevalent CVD among adults with diabetes in the clinical cohort.

In the clinical cohort, significant positive associations between HbA1c and prevalent CVD were observed among participants aged 40–59 years (OR, 1.634; 95% CI, 1.421–1.879; *P* < 0.001) and 60–79 years (OR, 1.307; 95% CI, 1.155–1.479; *P* < 0.001), with a significant interaction between HbA1c and age group (*P* for interaction=0.003). No significant interactions were observed for sex, BMI category, smoking status, drinking status, or hypertension (all *P* for interaction>0.05). Given the exploratory nature of the subgroup analyses and the limited number of CVD events in some strata, particularly the single CVD case in the 20–39-year age group of the clinical cohort, these findings should be interpreted cautiously.

### 3.4 Potential nonlinear associations between glycemic biomarkers and prevalent CVD assessed by RCS

RCS analyses were performed to examine potential nonlinear associations between glycemic biomarkers and prevalent CVD among adults with diabetes (**Figure 4**). In the NHANES cohort, HbA1c showed a significant overall association with prevalent CVD (*P*-overall = 0.027), without evidence of nonlinearity (*P*-nonlinear = 0.520). In contrast, TyG showed a significant nonlinear association with prevalent CVD (*P*-nonlinear <0.001), whereas FBG was not significantly associated with prevalent CVD. In the clinical cohort, HbA1c showed a significant nonlinear association with prevalent CVD (*P*-nonlinear = 0.009), whereas neither FBG nor TyG showed significant associations. Detailed knot locations, reference values, and statistical tests are provided in **Supplementary Table 2**.

**Figure 4:**
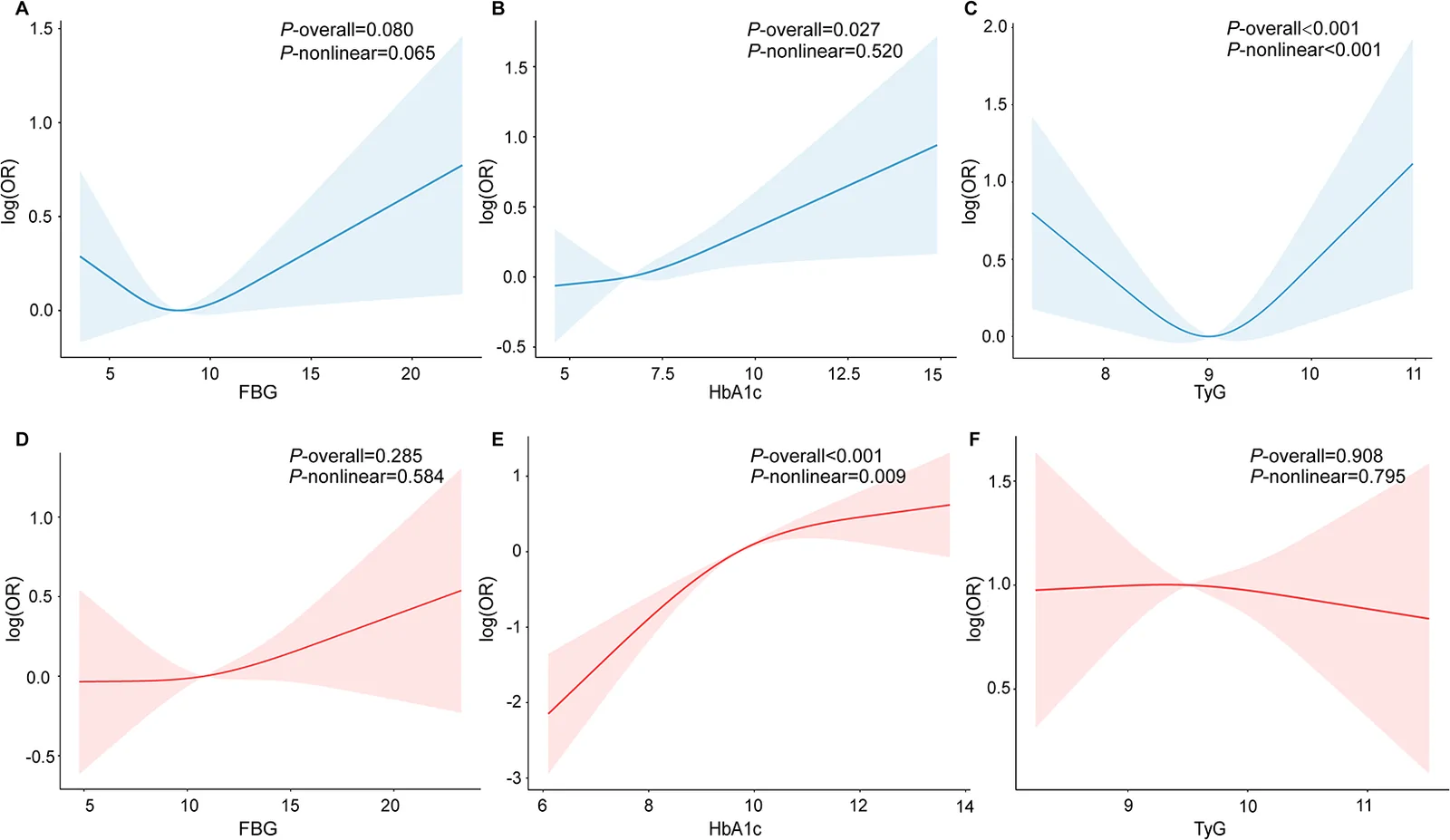
RCS plots adjusted for covariates. **(A)** FBG and CVD in NHANES; **(B)** HbA1c and CVD in NHANES; **(C)** TyG and CVD in NHANES; **(D)** FBG and CVD in clinical cohort; **(E)** HbA1c and CVD in clinical cohort; **(F)** TyG and CVD in clinical cohort. In the NHANES cohort, models were adjusted for age, sex, Race, PIR, smoking status, alcohol consumption, BMI, hypertension, TC, HDL-C, LDL-C, eGFR, glucose-lowering medication use, insulin use, lipid-lowering medication use, antihypertensive medication use, and Hb. In the clinical cohort, models were adjusted for age, sex, smoking status, alcohol consumption, BMI, hypertension, TC, HDL-C, LDL-C, eGFR, glucose-lowering medication use, insulin use, lipid-lowering medication use, antihypertensive medication use, Hb, and diabetes duration. Race/ethnicity and PIR were not included in the clinical cohort models because the clinical cohort consisted exclusively of Han Chinese participants and did not have standardized socioeconomic data. RCS, restricted cubic spline; CVD, cardiovascular disease; FBG, fasting blood glucose; HbA1c, glycated hemoglobin A1c; TyG, triglyceride-glucose index; TC, total cholesterol; HDL-C, high-density lipoprotein cholesterol; LDL-C, low-density lipoprotein cholesterol; eGFR, estimated glomerular filtration rate; Hb, hemoglobin; PIR, poverty-to-income ratio.

## 4 Discussion

In this study, we examined the associations between glycemic biomarkers and prevalent CVD among adults with diabetes using two independent cross-sectional cohorts, including a population-based survey and a clinical cohort. HbA1c showed a positive association with prevalent CVD in both cohorts, although the patterns observed in the RCS analyses differed between the two populations: no evidence of nonlinearity was observed in the NHANES cohort, whereas a nonlinear association was identified in the clinical cohort. In contrast, associations of FBG and TyG with prevalent CVD were not consistent across the two cohorts.

The increased burden of CVD among individuals with diabetes involves multiple interconnected mechanisms, including chronic hyperglycemia, insulin resistance, oxidative stress, endothelial dysfunction, and systemic inflammation (29–31). Persistent hyperglycemia has been implicated in vascular injury through the formation of advanced glycation end products, activation of oxidative pathways, and impairment of endothelial function (32, 33). As an indicator of long-term glycemic exposure, HbA1c reflects average glycemic status over several months and may provide a more stable measure of glycemic exposure than a single blood glucose measurement (34). In contrast, FBG reflects glycemic status at a single time point and may be affected by short-term biological variation, whereas TyG is a surrogate marker of insulin resistance that incorporates both glucose and triglyceride concentrations (35). These differences in the biological information captured by the biomarkers may partly contribute to the more consistent association observed for HbA1c across the two cohorts.

Previous studies have reported associations between glycemic parameters and cardiovascular outcomes in individuals with prediabetes and diabetes, but comparisons of different glycemic indicators across distinct diabetic populations remain limited (35–37). In the present study, HbA1c showed a consistent positive association with prevalent CVD in both cohorts, whereas the shape of the association differed between the population-based and clinical samples. The absence of significant nonlinearity for HbA1c in the NHANES cohort and the nonlinear association observed in the clinical cohort may reflect differences in the distributions of glycemic levels, disease severity, treatment patterns, and other clinical characteristics between the two populations. These findings should therefore be interpreted in the context of the distinct characteristics of the two cohorts rather than as evidence of a specific biological threshold.

In addition, TyG showed a significant nonlinear association with prevalent CVD in the NHANES cohort, whereas FBG was not significantly associated with prevalent CVD. TyG incorporates both glucose and triglyceride concentrations and is commonly used as a surrogate marker of insulin resistance (35). The nonlinear association observed for TyG may reflect the complex relationship between insulin resistance, metabolic dysfunction, and CVD across different levels of glycemic and lipid status. In contrast, FBG represents glycemic status at a single time point and may be influenced by short-term biological variation, which may partly explain the absence of a significant association in the present analysis. However, these potential explanations remain speculative, and the observed nonlinear pattern for TyG should be interpreted cautiously given the cross-sectional design and differences in metabolic characteristics between the two cohorts. Longitudinal studies are needed to determine whether these associations persist over time and to clarify their temporal relationships with CVD.

From a clinical perspective, the consistent association between HbA1c and prevalent CVD across the two cohorts supports the relevance of long-term glycemic exposure when evaluating CVD burden among individuals with diabetes. However, because of the cross-sectional design, these findings should not be interpreted as evidence that HbA1c predicts future CVD events or that modifying HbA1c would necessarily reduce cardiovascular risk. The subgroup analyses did not provide consistent evidence of effect modification across demographic and clinical characteristics, and the significant age interaction observed in the clinical cohort should be interpreted cautiously given the limited number of CVD events in some age strata, particularly the single CVD case among participants aged 20–39 years.

This study has several limitations. First, the cross-sectional design precludes establishing temporality between glycemic biomarkers and CVD and does not allow causal inference. Reverse causation is also possible, as pre-existing CVD or its associated physiological and treatment-related changes may influence glycemic measures. Second, although we adjusted for a broad range of demographic, lifestyle, metabolic, renal, hematological, and medication-related covariates, residual confounding from unmeasured or incompletely measured factors cannot be excluded. Third, the ascertainment of CVD differed between the two cohorts. In NHANES, CVD status was based on standardized self-reported physician diagnoses, whereas the clinical cohort used documented medical records and clinical diagnoses. Although the same major cardiovascular conditions were considered in both cohorts, differences in ascertainment may have introduced outcome misclassification and may partly contribute to the observed differences between populations. Fourth, the NHANES analysis was restricted to participants with available fasting biomarker and covariate data. Although this approach was necessary for analyses involving fasting laboratory measurements, exclusion of participants who did not meet the fasting requirements or had missing data may have introduced selection bias, and this possibility cannot be completely excluded. Fifth, the clinical cohort was recruited from a single hospital and involved predefined exclusion criteria, including cancer, inflammatory diseases, and severe renal or hepatic dysfunction. These selection criteria may have resulted in a highly selected study population, limiting the generalizability of the findings to other populations and healthcare settings and potentially introducing selection bias or collider bias. Finally, differences in glucose-lowering therapy, insulin use, lipid-lowering therapy, and antihypertensive treatment between individuals with and without CVD may reflect both clinical management and underlying disease severity, making it difficult to fully separate treatment effects from disease-related factors in a cross-sectional analysis. Longitudinal, multicenter studies with harmonized outcome ascertainment and comprehensive covariate assessment are needed to clarify the temporal relationships between glycemic biomarkers and CVD.

## 5 Conclusion

In this study, we evaluated the associations between multiple glycemic biomarkers and prevalent CVD among adults with diabetes using two independent cross-sectional cohorts. HbA1c was consistently and positively associated with prevalent CVD in both the population-based and clinical cohorts, whereas the associations of FBG and TyG with prevalent CVD were less consistent across populations. Although the shape of the HbA1c–CVD association differed between cohorts, the consistent association observed for HbA1c suggests that long-term glycemic status is relevant to the burden of prevalent CVD among individuals with diabetes. Further longitudinal studies are warranted to clarify the temporal relationship between HbA1c and CVD and to determine whether these findings extend to future cardiovascular outcomes.

## Data Availability

The raw data supporting the conclusions of this article will be made available by the authors, without undue reservation.
