# Supplementary material for: Glycemic biomarkers and cardiovascular disease in diabetes: a comparative analysis of population-based and clinical cohorts
Source: Front Endocrinol (Lausanne). 2026 Oct 14;17:1995413. doi: 10.3389/fendo.2026.1995413 (PMC13713321; doi:10.3389/fendo.2026.1995413)
Supplement: Supplementary file 1 [file Table1.docx]

**Supplementary Table 1** Associations of standardized glycemic biomarkers with prevalent CVD in the fully adjusted models.

| Model | NHANES | |  | Clinical cohort | |  |
| --- | --- | --- | --- | --- | --- | --- |
|  | Standardized OR (95% CI) | *P* |  | Standardized OR (95% CI) | *P* |  |
| **Model 4** |  |  |  |  |  |  |
| FBG | 1.077 (0.933, 1.242) | 0.310 |  | 1.138 (0.961, 1.349) | 0.136 |  |
| HbA1c | 1.175 (1.004, 1.376) | 0.045 |  | 1.944 (1.608, 2.364) | <0.001 |  |
| TyG | 0.981 (0.742, 1.298) | 0.894 |  | 0.970 (0.817, 1.149) | 0.723 |  |

**Notes:** ORs and 95% CIs were estimated per 1-SD increase in each biomarker using the fully adjusted model (Model 4). In the NHANES cohort, Model 4 was adjusted for age, sex, Race, PIR, smoking status, drinking status, BMI, hypertension, TC, HDL-C, LDL-C, eGFR, glucose-lowering medication use, insulin use, lipid-lowering medication use, antihypertensive medication use, and Hb. In the clinical cohort, Model 4 additionally included diabetes duration. Race/ethnicity and PIR were not included in the clinical cohort model because the cohort consisted exclusively of Han Chinese participants and did not have standardized socioeconomic data. Standardized values were calculated separately within each cohort.

**Abbreviations:** CVD, cardiovascular disease; FBG, fasting blood glucose; HbA1c, glycosylated hemoglobin A1c; TyG, triglyceride-glucose index; PIR, poverty-to-income ratio; BMI, body mass index; TC, total cholesterol; HDL-C, high-density lipoprotein cholesterol; LDL-C, low-density lipoprotein cholesterol; eGFR, estimated glomerular filtration rate; Hb, hemoglobin; SD, standard deviation; OR, odds ratio; CI, confidence interval.

| Cohort | Biomarker | Knot1 | Knot2 | Knot3 | Reference | *P*-overall | *P*-nonlinear |
| --- | --- | --- | --- | --- | --- | --- | --- |
| NHANES | FBG | 5.61 | 7.22 | 12.30 | 7.22 | 0.080 | 0.065 |
| NHANES | HbA1c | 5.60 | 6.60 | 9.30 | 6.60 | 0.027 | 0.520 |
| NHANES | TyG | 8.24 | 9.03 | 9.90 | 9.03 | <0.001 | <0.001 |
| Clinical cohort | FBG | 6.57 | 10.83 | 16.69 | 10.83 | 0.285 | 0.584 |
| Clinical cohort | HbA1c | 7.00 | 9.70 | 12.26 | 9.70 | <0.001 | 0.009 |
| Clinical cohort | TyG | 8.74 | 9.51 | 10.51 | 9.51 | 0.908 | 0.795 |

**Supplementary Table 2** Parameters and statistical tests of RCS analyses for the associations between glycemic biomarkers and prevalent CVD.

**Notes:** RCS analyses were performed using three knots for each glycemic biomarker. Knot locations were determined within the restricted cubic spline model, and the median value of each biomarker was used as the reference value.

**Abbreviations:** RCS, restricted cubic spline; CVD, cardiovascular disease; FBG, fasting blood glucose; HbA1c, glycated hemoglobin A1c; TyG, triglyceride-glucose index.
